# Intraoperative myocardial infarction and refractory cardiogenic shock during major hepatectomy: a case report

**DOI:** 10.1186/s40981-022-00510-x

**Published:** 2022-03-09

**Authors:** Yasunori Yagi, Kazuyuki Mizunoya, Toshihiro Mori, Hitoshi Saito, Yuji Morimoto

**Affiliations:** 1 Department of Anesthesiology and Critical Care Medicine, Hokkaido University Hospital, N14, W5, Kita-ku, Sapporo, 060-8648 Japan; 2 Department of Anesthesiology, Sapporo City Hospital, N11, W13, Chuo-ku, Sapporo, 060-8604 Japan

**Keywords:** Myocardial infarction, Hepatectomy, Cardiogenic shock, Transesophageal Echocardiography

## Abstract

**Background:**

Myocardial infarction (MI) complicated by cardiogenic shock during non-cardiac surgery is a rare but fatal complication. The management of intraoperative MI is challenging.

**Case presentation:**

A 77-year-old hypertensive man with good functional capacity was scheduled for hepatectomy. After the start of liver resection, the electrocardiogram monitor showed ST depression, and the patient developed refractory cardiogenic shock. Transesophageal echocardiography revealed severe hypokinesis of the anteroseptal wall. The surgery was suspended, and an intra-aortic balloon pump was placed following immediate abdominal closure. Coronary angiography revealed severe stenosis of the left main coronary trunk, and percutaneous coronary intervention (PCI) was performed. Myocardial wall motion improved, and blood pressure stabilized. Two days after PCI, hepatectomy, which had been suspended, was successfully completed.

**Conclusions:**

Once intraoperative MI has occurred, early diagnosis and multidisciplinary approaches are important to manage the difficult clinical situation.

## Background

Myocardial infarction (MI) complicated by cardiogenic shock (CS) during non-cardiac surgery is a rare but life-threatening complication. Proper preoperative assessment of cardiovascular risk is important for its prevention. Although there has been a focus on the prevention of perioperative MI (PMI) in recent reviews and guidelines [1–3], few studies exist on the management of PMI, especially intraoperative MI. Once intraoperative MI has occurred, the management must be decided in consideration with factors such as the surgical procedure and progress, the bleeding risk, severity of MI, and hemodynamics. Herein, we describe the anesthetic management of a case of MI with refractory CS during hepatectomy on a patient with hypertension and good functional capacity.

## Case presentation

A 77-year-old hypertensive man (height, 179.2 cm; weight, 71.4 kg) was scheduled for elective extended right hepatectomy for cholangiocarcinoma. Although the patient was elderly, he could ski asymptomatically. The preoperative electrocardiogram was normal, and no further cardiovascular examinations were performed.

**Fig. 1:**
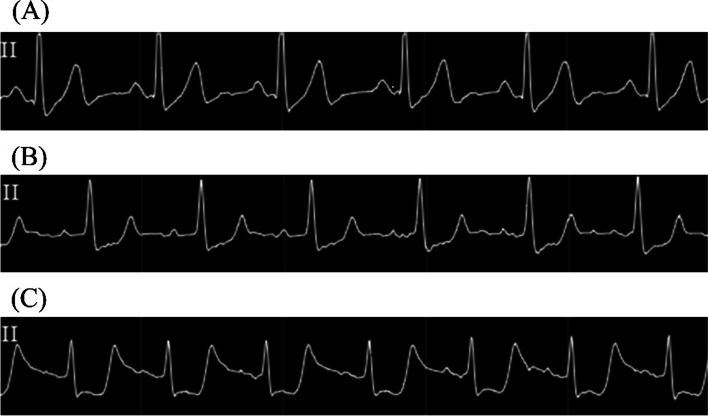
The electrocardiogram monitor **A** before anesthesia induction, **B** after the start of liver resection, and **C** at the onset of cardiogenic shock. The electrocardiogram monitor showed slow upsloping ST depression at the start of liver resection and horizontal ST depression at the onset of cardiogenic shock in lead II

**Fig. 2:**
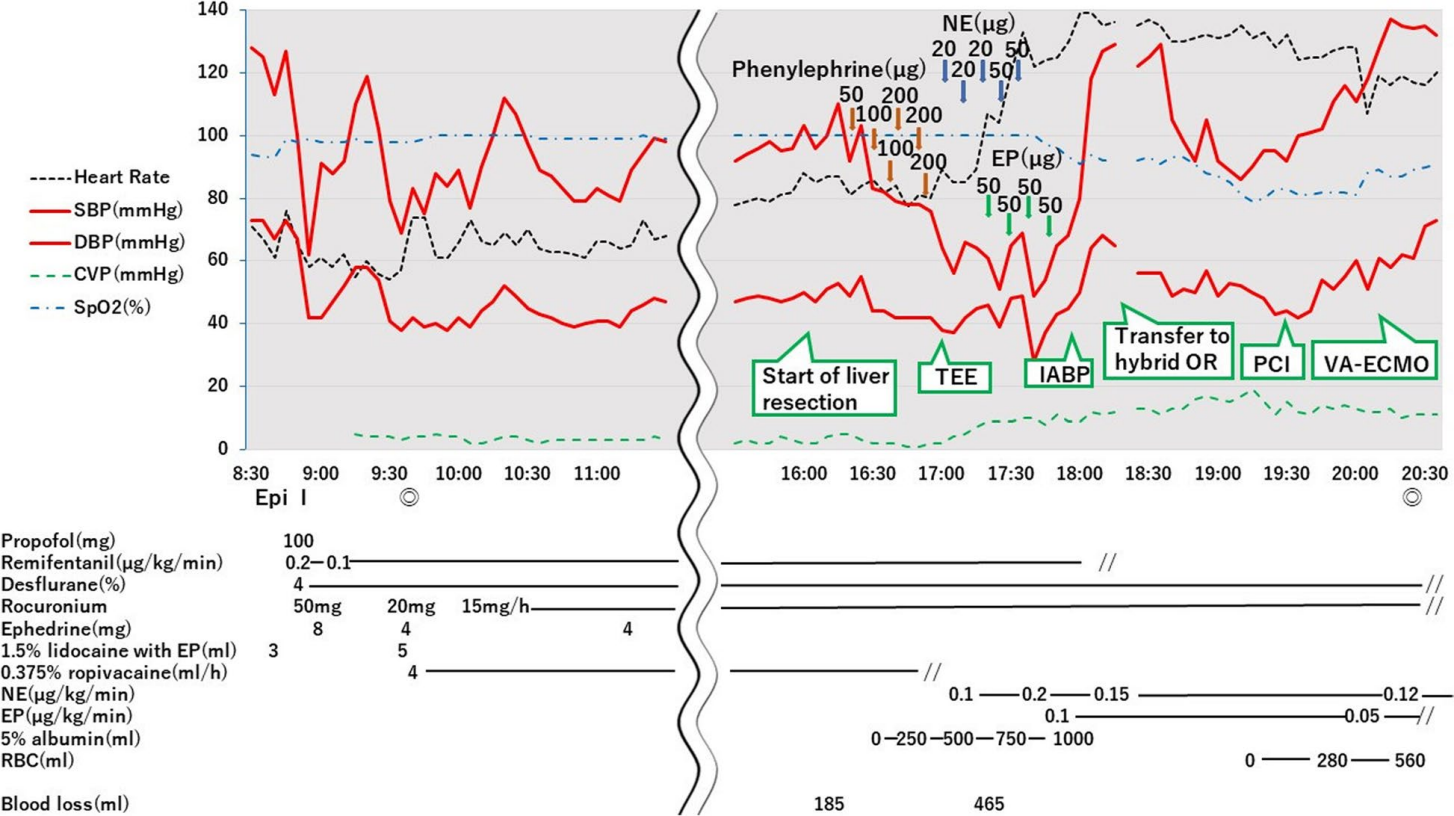
Anesthesia record. 1.5% lidocaine with EP and 0.375% ropivacaine were administered epidurally. Epi, epidural anesthesia; I, intubation; ◎, surgery start and end. SBP, systolic blood pressure; DBP, diastolic blood pressure; CVP, central venous pressure; SpO_2_, peripheral oxygen saturation; NE, norepinephrine; EP, epinephrine; TEE, transesophageal echocardiography; IABP, intra-aortic balloon pump; OR, operating room; PCI, percutaneous coronary intervention; VA-ECMO, veno-arterial extracorporeal membrane oxygenation

Before anesthesia induction, a 3-lead electrocardiogram (ECG) showed rapid upsloping ST depression in lead II (Fig. 1A), but the patient did not complain of any ischemic symptoms. Following epidural catheter insertion at the level of Th8–Th9, general anesthesia was induced with propofol, rocuronium, fentanyl, and remifentanil and maintained with desflurane. After a smooth intubation, a right radial arterial catheter and a central venous catheter were placed. Vital signs before liver resection were stable (Fig. 2), and fluid administration was restricted to maintain a low central venous pressure (CVP) to reduce blood loss during liver resection.

**Fig. 3:**
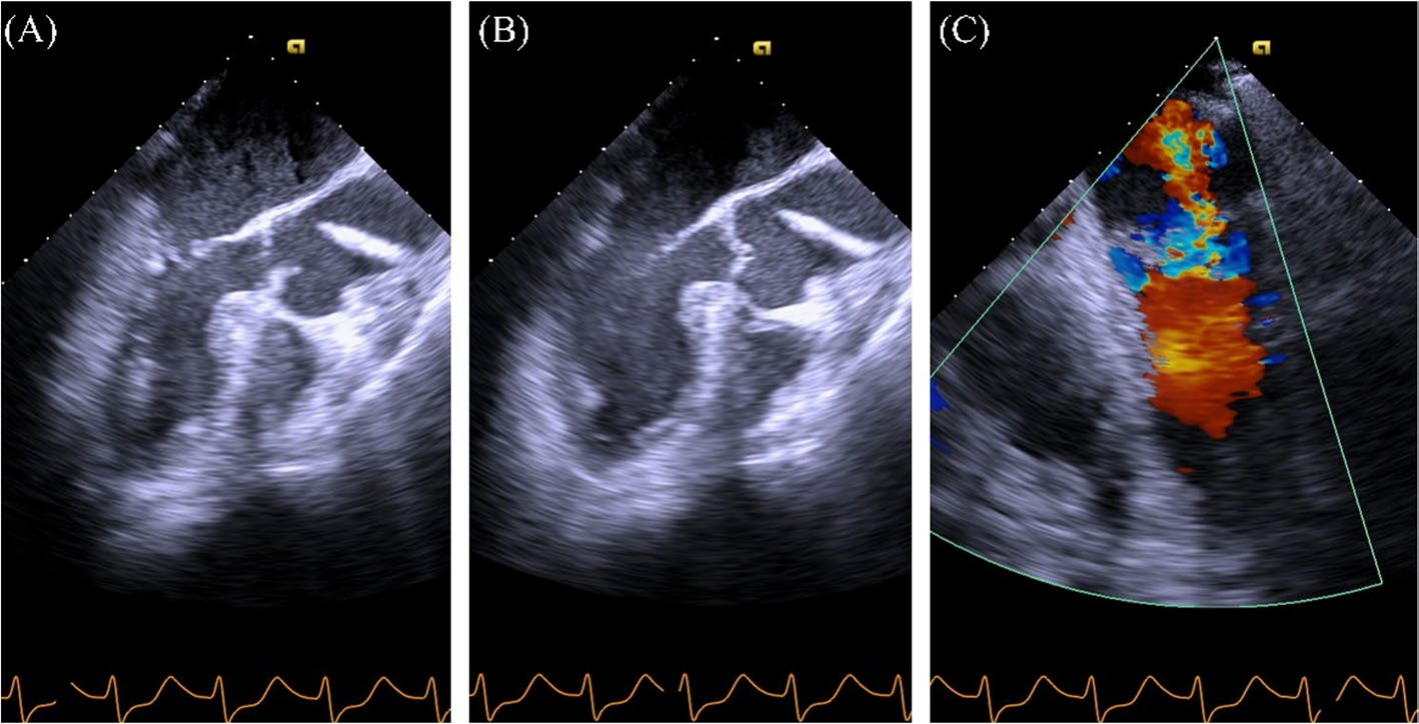
Intraoperative transesophageal echocardiography(TEE). **A**, **B** TEE images showing severe hypokinesis of the anteroseptal wall in **A** (systole) and **B** (diastole) (midesophageal long-axis view). **C** A TEE image with the application of color Doppler showing severe mitral regurgitation (midesophageal four-chamber view)

**Fig. 4:**
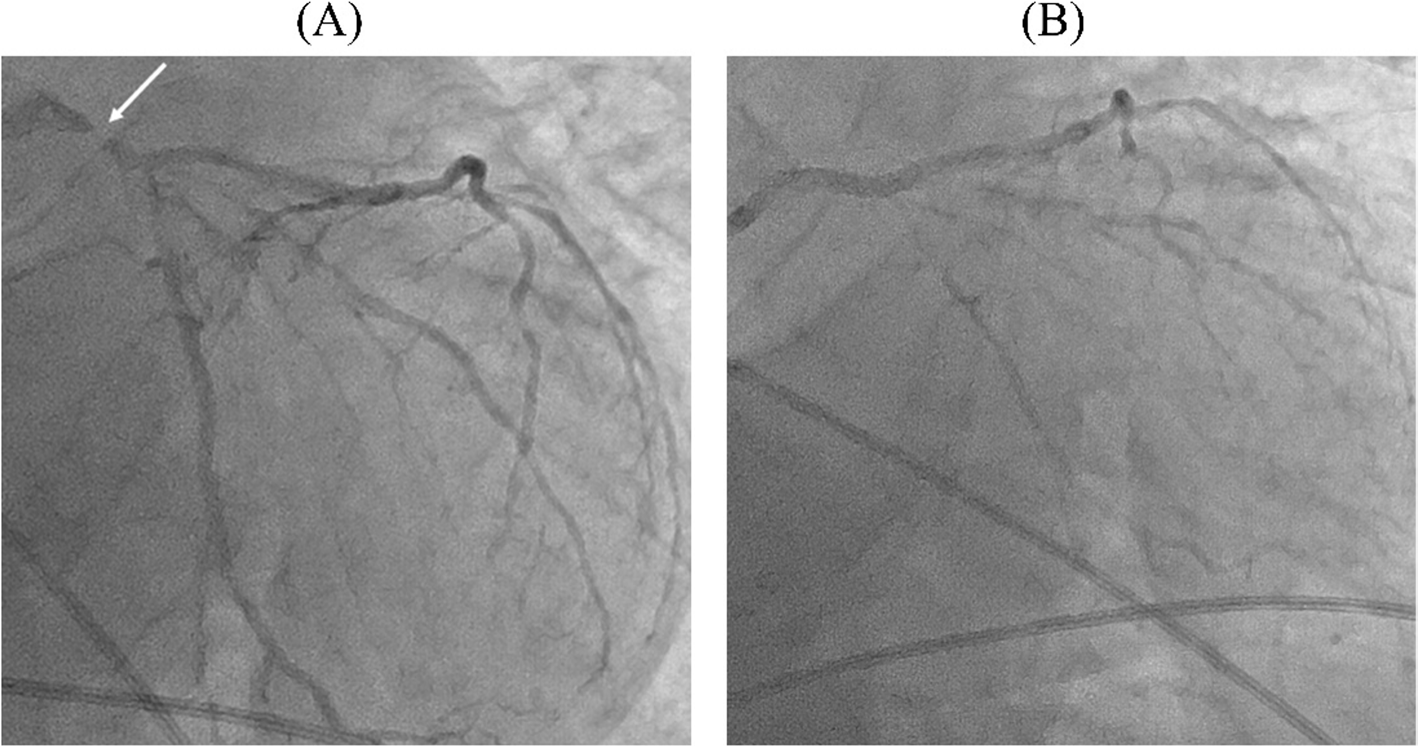
Coronary angiography (CAG). **A** CAG in the right anterior oblique position showing severe stenosis of the mid-left main coronary trunk (LMT) (arrow). **B** The final CAG in the right anterior oblique position showing optimal dilatation of the LMT

After the start of liver resection, the mean arterial pressure (MAP) decreased to 50 mmHg (Fig. 2), and ECG showed slow upsloping ST depression in lead II (Fig. 1B). The authors speculated that this hypotension occurred due to bleeding and compression of the inferior vena cava by the surgeon. Therefore, multiple doses of phenylephrine and a bolus of 5% albumin solution were administered. Despite these measures, hypotension with a MAP of 50–60 mmHg persisted for approximately 30 min. The management of hypotension became gradually difficult, and MAP decreased to a nadir of 36 mmHg. Subsequently, multiple doses of norepinephrine and epinephrine were administered, followed by continuous infusion. The patient did not respond to this, and ECG showed horizontal ST depression in lead II (Fig. 1C). Transesophageal echocardiography (TEE)—performed to diagnose the cause of refractory hypotension—revealed severe hypokinesis of the anteroseptal wall, a left ventricular ejection fraction (LVEF) of 20%, and severe mitral regurgitation (MR) (Fig. 3). We considered the diagnosis of CS owing to the occurrence of MI intraoperatively; the surgery had to be suspended in the middle of the parenchymal resection. An intra-aortic balloon pump (IABP) was placed following immediate abdominal closure, and the patient was transferred to a nearby hybrid operating room for coronary angiography (CAG). Blood sampling at this time revealed 273 ng/L of troponin-I and 10.1 g/dL of hemoglobin. CAG revealed severe stenosis of the left main coronary trunk (LMT) (Fig. 4A). An intravascular ultrasound study (IVUS) revealed the presence of a stenotic lesion with ulceration in the mid-portion of the LMT. An emergent PCI was performed, and the final CAG showed optimal dilatation of the LMT stent (Fig. 4B). Myocardial wall motion and MR improved, and blood pressure stabilized, but oxygen saturation decreased to a nadir of 76% (FiO_2_ 100%) due to pulmonary edema. Additionally, an elevated CVP of up to 20 mmHg caused severe bleeding in the resection plane of the liver. Therefore, femoro-femoral veno-arterial extracorporeal membrane oxygenation (VA-ECMO) was initiated to reduce organ congestion, and the patient was transferred to the intensive care unit. During VA-ECMO, unfractionated heparin was infused at 200–400 units/h to maintain an activated clotting time of 160–200 s. The patient was weaned off VA-ECMO after improvement of the pulmonary edema, 18 h later. Hepatectomy, which had been suspended, was successfully completed after 36 h with the support of IABP and infusions of norepinephrine (0.05 μg/kg/min), dobutamine (5 μg/kg/min), and landiolol (2 μg/kg/min). The patient bled continuously for 36 h (volume 6500 mL) till the reoperation, owing to the hemi-resected liver parenchyma and heparin administration for VA-ECMO. Twenty units of packed red blood cells, 38 units of frozen fresh plasma, and 60 units of platelet concentrates were transfused. After reoperation, the continuous bleeding improved. The IABP was removed on postoperative day (POD) 4 of the initial surgery, and the trachea was extubated on POD 7. Although the patient died due to sepsis during the course of treatment for postoperative liver failure on POD 90, he remained free from any other cardiovascular events during hospitalization.

## Discussion

Based on the available literature [4–6], the incidence of PMI is 0.6 to 5% depending on the target population, study design, and the PMI definition. PMI itself is not a rare complication; however, intraoperative MI complicated by refractory CS during non-cardiac surgery, as in this case, is rare. MI that develops during hepatectomy has a high risk of treatment-related bleeding, which complicates the management.

Proper preoperative assessment of cardiovascular risk is important for the prevention of PMI [1, 2]. The guidelines on perioperative cardiovascular evaluation and management published by the American College of Cardiology/American Heart Association (ACC/AHA) [1] and European Society of Cardiology/European Society of Anaesthesiology [2] have proposed a stepwise approach to perioperative cardiac assessment, including the type of surgery, comorbidities, cardiac conditions, and functional capacity. In this case, although hepatectomy was a high-risk surgery, the patient had no other risk factors as defined by the Revised Cardiac Risk Index. Furthermore, he was able to ski asymptomatically, which was equivalent to 7.5 metabolic equivalents [1]. Therefore, following the guidelines, we proceeded with the planned surgery without further cardiovascular testing. Preoperative evaluation according to these guidelines could not reveal the risk of cardiovascular complications in this case.

Although the etiology of PMI is incompletely understood, coronary thrombosis (classified as type 1 PMI) and supply-demand mismatch (classified as type 2 PMI) [7] appear to be important causes. In this case, mild hypotension and slow upsloping ST depression occurred after the start of the liver resection, which persisted for approximately 30 min, after which the patient developed refractory CS. This clinical course led us to speculate that the etiology of MI in this case was a supply-demand mismatch caused by hypotension. Therefore, we administered noradrenaline and adrenaline to raise coronary perfusion pressure instead of administering a coronary dilator. We did not administer nitrates in this case, as the guidelines [8, 9] recommend avoidance of nitrates in patients with hypotension. In fact, IVUS revealed the presence of a stenotic lesion with ulceration, which strongly suggested type 1 PMI. Intraoperative factors such as inflammation, hypercoagulability, and blood pressure fluctuations might have caused plaque rupture and acute subtotal occlusion. Moreover, persistent hypotension further exacerbated myocardial ischemia, which caused left ventricular asynergy followed by functional MR, resulting in a vicious cycle of profound hypotension and coronary ischemia, leading to refractory CS.

Many recent reviews and guidelines on PMI focus on its prevention [1–3], but there are a few studies on the management of PMI, especially intraoperative MI. Once intraoperative MI has occurred, the management must be decided in consideration with factors such as surgical procedure, surgical progress, bleeding risk, the severity of MI, and hemodynamics. Early diagnosis and multidisciplinary approaches are important for managing this difficult clinical situation. We immediately performed TEE to investigate the etiology of CS and decided to insert IABP and perform CAG because conservative treatment could not improve the situation. Although routine use of IABP in patients with acute MI (AMI) complicated by CS is not recommended [10], in this case, immediate IABP insertion improved hemodynamics by coronary artery perfusion improvement and left ventricle afterload reduction. This hemodynamic improvement by IABP enabled the patient to be safely transferred to the hybrid operating room.

In general, invasive treatment for PMI has a risk of bleeding; conservative treatment is, therefore, preferred [3, 11]. In fact, Smithowitz et al. reported that only 23.6% of perioperative AMI patients were treated invasively, even among patients with STEMI [4]. However, this patient developed refractory CS, and it seems that the patient could not have been saved without invasive treatment. Despite the extremely high risk of bleeding from the hemi-resected liver parenchyma, this case was successfully treated with invasive treatment with proper transfusion management. Many studies [12–14] have revealed that early revascularization reduces mortality in patients with CS complicating AMI. As with AMI, early revascularization seems to be important for patients with intraoperative MI complicated by CS, even if the bleeding risk is high.

In summary, we described a case of MI with CS during hepatectomy. Anesthesiologists should recognize that intraoperative MI can occur, even for cases evaluated as “reasonable to proceed with planned surgery without further cardiovascular testing” by the relevant guidelines. Once intraoperative MI has occurred, early diagnosis and multidisciplinary approaches are important. Despite the extremely high risk of bleeding during liver resection, the patient was successfully treated with invasive treatment and proper transfusion management.

## Data Availability

Not applicable

## Abbreviations

MI: Myocardial infarction
CS: Cardiogenic shock
PMI: Perioperative myocardial infarction
PCI: Percutaneous coronary intervention
IABP: Intra-aortic balloon pump
TEE: Transesophageal echocardiography
LVEF: Left ventricular ejection fraction
MR: Mitral regurgitation
CAG: Coronary angiography
LMT: Left main coronary trunk
IVUS: Intravascular ultrasound study
VA-ECMO: Veno-arterial extracorporeal membrane oxygenation
POD: Postoperative day
RCRI: Revised Cardiac Risk Index
